# Available Technologies, Applications and Benefits of Teleorthodontics. A Literature Review and Possible Applications during the COVID-19 Pandemic

**DOI:** 10.3390/jcm9061891

**Published:** 2020-06-17

**Authors:** Cinzia Maspero, Andrea Abate, Davide Cavagnetto, Mohamed El Morsi, Andrea Fama, Marco Farronato

**Affiliations:** 1 Department of Biomedical, Surgical and Dental Sciences, School of Dentistry, University of Milan, 20100 Milan, Italy; andreabate93@gmail.com (A.A.); davide.cavagnetto@gmail.com (D.C.); moh.nagi90@gmail.com (M.E.M.); andreas.fama@hotmail.it (A.F.); marco.farronato@unimi.it (M.F.); 2 Fondazione IRCCS Cà Granda, Ospedale Maggiore Policlinico, 20100 Milan, Italy

**Keywords:** COVID-19, dentistry, teleassistance, remote sensing technology, orthodontics, teledentistry, teleorthodontics

## Abstract

Background: COVID-2019 spread rapidly throughout the world from China. This infection is highly contagiousness, has a high morbidity, and is capable of evolving into a potentially lethal form of interstitial pneumonia. Numerous countries shut-down various activities that were considered “not essential.” Dental treatment was in this category and, at the time of writing, only non-deferrable emergencies are still allowed in many countries. Therefore, follow-up visits of ongoing active therapies (e.g., orthodontic treatment) must be handled taking special precautions. This literature review aims at reducing in-office appointments by providing an overview of the technologies available and their reliability in the long-distance monitoring of patients, i.e., teledentistry. Methods: A literature review was made according to Preferred Reporting Items for Systematic Reviews and Meta-Analyses Protocols (PRISMA-P) guidelines. Randomized clinical trials, cross sectional, observational, and case-control studies were evaluated with the Mixed Methods Appraisal Tool for quality assessment and study limitations. Results: A primary search found 80 articles, 69/80 were excluded as non-relevant on the basis of: the abstract, title, study design, bias, and/or lack of relevance. Twelve articles were included in the qualitative analysis. Conclusions: Teleorthodontics can manage most emergencies, reassuring and following patients remotely. The aim set by dental teleassistance was met as it reduced patients’ office visits whilst maintaining regular monitoring, without compromising the results. Although our preliminary findings should be further investigated to objectively evaluate the efficacy, cost-effectiveness, and long-term results, we are confident that teleassistance in orthodontics will have a role to play in the near future.

## 1. Introduction

A new type of coronavirus initially named Novel Coronavirus Pneumonia (NCP) and later renamed new Corona Virus 2019 (2019-nCoV or Covid-19) spread rapidly from China to the world from December 2019. It is the seventh coronavirus known to spillover to humans [1]. This viral infection is of great concern due to its high contagiousness and morbidity, as well as its ability to evolve into a potentially lethal form of interstitial pneumonia and its possible evolution into a potentially lethal form of interstitial pneumonia [2]. Preventive hygiene measures such as social distancing, quarantine, and isolation have been taken to limit its diffusion in most countries to different extent [3]. On 30 January 2020, the World Health Organization (WHO) stated that COVID-19 constituted a public health emergency of international relevance [4]. 

The National Health Committee keeps receiving an ever-increasing number of confirmed, suspected, and fatal cases reported from all over the world. To date, they are still carrying out world surveillance. 

There was an estimated human-to-human healthcare-related transmission of about 41% at the beginning of the outbreak [5]. Many health care workers got and still are getting infected [5].

Government and healthcare services have put on their thinking hats to re-organize triage services in an attempt to reduce nosocomial infection by COVID 19 [5]. This task is particularly arduous as transmission is mainly through droplets and numerous subjects may be asymptomatic and/or in the incubation period. Dental clinics belong to a high-risk category as infection can be facilitated during dental maneuvers that generate droplets, including restorative procedures, professional hygiene sessions, etc., or whilst patients are in the waiting room [6]. Therefore, strict and effective hygiene protocols for infection control are urgently needed for dental practices to reduce dental practitioners’ and patients’ risk to get infected.

The use of appropriate personal protective equipment (PPE) is pivotal in avoiding cross infection during clinical practice between patients and healthcare workers and the adoption of adequate decontamination measures can help to reduce the risks. Although it has also currently been suggested that dental clinics limit their practice only to not deferrable emergencies, this is not always possible. Some ongoing treatment such as orthodontic therapies and/or critical situations, like conditions that must be identified in the early stages and treated immediately to avoid more serious outcomes, require timely follow-up appointments. Indeed, continuous monitoring by the orthodontist is a must in orthodontic treatment so as to evaluate the efficacy and/or any undesirable effects [7,8]. 

However, some periodic visits are not strictly necessary and others could be delayed by instructing the patient how to make simple changes to the appliance, for example by indicating which teeth to put the intraoral elastics on or how many activations to perform on the central screw of a rapid palatal expander.

At the time of writing, despite huge investments and research efforts, the current pandemic is still under investigation as are the best preventive measures to be adopted in individual fields. However, we are of the opinion that avoiding unnecessary follow-up appointments whilst maintaining the monitoring of treatment outcomes and current health status would be of great interest and importance for healthcare providers. Recently, an innovative approach has been proposed in the medical field. Although it was originally developed to provide healthcare services in remote areas, it may well be of use in managing healthcare services in this unprecedented emergency situation, i.e., telemedicine. 

The World Health Organization (WHO) defines telemedicine as the use of telecommunications and virtual technologies to provide healthcare outside of traditional healthcare facilities [9]. In more detail, telemedicine is a set of technologies, especially Information and Communication Technologies (ICT), specifically aimed at providing healthcare services from a distance to lessen the need for contact between the patient and the healthcare provider [10,11] Secure communication of medical information, notes, sounds, pictures, or any other form of data necessary are required to prevent, or to diagnose pathologies, and therefore, to treat and to monitor patients [9]. 

Moreover, telemedicine is not only able to facilitate communication and interaction between the healthcare provider and the patient, but also between the providers themselves. Indeed, it can, to a certain extent, remove geographical and temporal barriers, bridging gaps in the dishomogeneous distribution of the healthcare offer. Therefore, it can provide care for more people, enabling them to benefit from healthcare services, especially those who live in remote areas and/or have poorly developed healthcare facilities. It can simplify online transmission of diagnostic tests and reduce waiting lists for consultations through an enhanced organization of appointments [12,13,14]. This makes these technologies a great resource in optimizing and reducing in-office visits and does not compromise necessary check-ups. Treatment progress and efficacy can be monitored in this time of social distancing, which will most likely be prolonged into the year to come as the international scientific community has declared that a definitive cure and/or vaccine is not yet available as research is still ongoing. Nowadays, telemedicine is becoming more and more widespread in the fields of oncology, cardiology, pediatrics, psychiatry, psychology, radiology, pneumology, dermatology, neurology, orthopedics, ophthalmology, and dentistry [15].

Although teleassistance in dentistry is far from new, it seems that its advantages in orthodontics have not yet been fully explored and is used on a limited scale. 

Indeed, there are some reviews on teledentistry in general but none on teleorthodontics as most articles about teleorthodontics are relatively new.

As no reviews have yet been carried out on the efficacy of teleassistance in orthodontics as a way to manage patients at a distance, we would like to report on the evidence available as to the possibility of implementing new technologies in teleassistance, generally known by teleorthodontics to help during the COVID-19 pandemic to remotely monitor patients’ conditions.

## 2. Experimental Section

### 2.1. Search Strategy

This topic is far from new, however, few studies have been reported, there is need for exploratory research for a better understanding. Given the above, a non-systematic literature review was performed [16]. The electronic literature was searched using the following databases: Medline, Pubmed, Embase, Cochrane Library, EBM Reviews, Web of Science, Ovid, and Google Scholar. The search was mainly based on five terms, i.e., teledentistry, teleorthodontics, virtual assistance, tele assistance, and telemedicine. Embase and PubMed were searched respectively using also the terms Embase subject headings (Emtree) and Medical Subject Heading (MeSH).

The EndNote software reference manager (Version X7 × 9.21, Thomson Reuters, released September 2014, Toronto, ON, Canada) was used/adopted to store/archive and view/analyze retrieved references studies. The research refers to the Preferred Reporting Items for Systematic Reviews and Meta-Analyses (PRISMA-P) 2015 [17,18]. Grey literature was also searched, but no data met the inclusion criteria. A hand search for relevant studies in the selected bibliography was also performed.

### 2.2. Inclusion Criteria 

Studies involving new or already existing devices and software for teleassistance in orthodontics were included. Service provided, type of intervention, clinical outcomes, efficacy and efficiency of assessed methods, and possible time saving compared to traditional methods were evaluated. The following study designs were included: observational studies, longitudinal studies, prospective studies, case-control studies, systematic and narrative reviews, and clinical trials. 

Given the state of technology and its rapid evolution, the search was limited to papers published over the previous 15 years.

### 2.3. Exclusion Criteria 

Studies in a language other than English or on application areas unrelated to orthodontics were excluded.

Articles with a poor methodology description lacking at least two of the following were excluded: study design, sample size, hardware utilized, software installed. Letters to the editor, short communications, and all other publications not subjected to the peer review process were also excluded.

### 2.4. Data Extraction and Quality Assessment

Considering the variety of study designs in the articles included, Mixed Methods Appraisal Tool (MMAT) was used for quality assessment [17]. The score of each article was calculated by dividing the criteria that were considered satisfied by 4 (25% by 1 criterion, 100% if all 4 criteria were considered satisfied). The use of this system is compatible with a literature review that analyzes different research methodologies, as reported by Whittemore [18].

Two of the authors of this study (A.F and M.E.M) read the titles of the retrieved articles independently to ensure they met the eligibility criteria. If in doubt, the abstracts were read and the same method was applied. A final selection was then made by an independent evaluation of the full text of aforementioned papers before inclusion. Any disagreement between the assessors was resolved by their discussing the full texts. The studies selected according to eligibility criteria are reported in the evidence table (Table 1). One reviewer (A.F) extracted data from the full-texts and the other (M.E.M), independently verified the extracted data. Data extraction included: journal and year of publication, study design, clinical outcomes, and the conclusions of the research. The description of the included studies is reported in Table 1.

### 2.5. Risk of Bias in Individual Studies

Due to the differences in the design of the selected studies, understandable given the heterogeneity of what was taken into account, it was not deemed fit to apply common methods for evaluation of the risk of bias. There was a low or absent overall risk of bias as to data description but high risk of bias for the efficacy analysis of such a technology. All considered papers had high or unknown selection bias and reference standards. Moreover, as this is a novel topic, to the best of our knowledge, no validated protocol has yet been reported.

### 2.6. Limitations of the Review

Due to the heterogeneity of study designs and the technological tools assessed, it was not possible to carry out a meta-analysis of the data. Therefore, a thematic investigation was made, targeting the main topics that were analyzed in the selected papers. No other limitations appear to be present, as the review was carried out according to the PRISMA-P guidelines.

## 3. Results 

**Figure 1:**
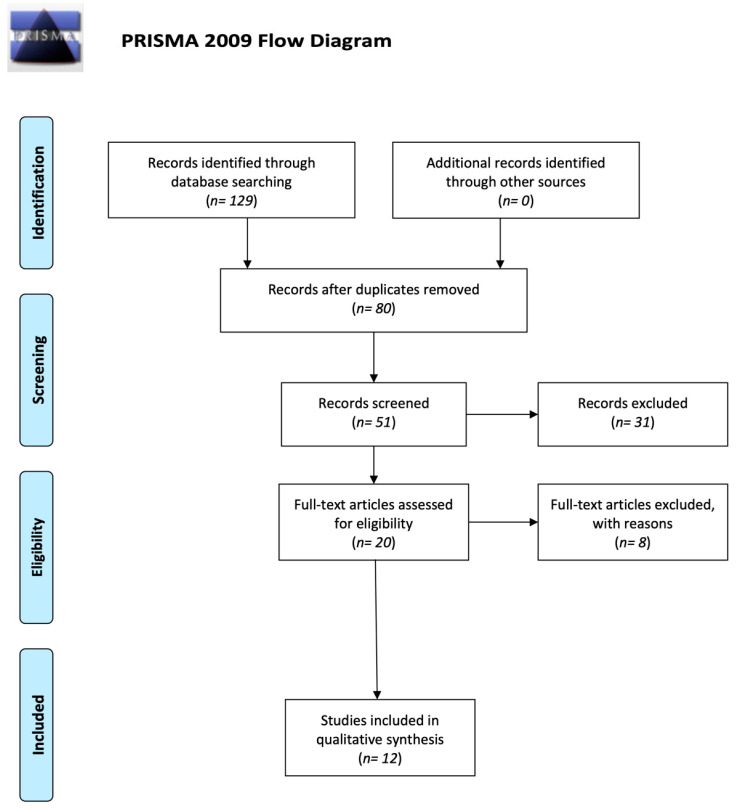
Preferred Reporting Items for Systematic Reviews and Meta-Analyses (PRISMA) flow chart.

Initially 129 articles were found. The primary search retrieved 80 articles, net of elimination of duplicates (*n* = 49). A total of 31 articles were then deemed irrelevant after screening the abstract, the title, or the study design and were therefore excluded. Twenty records were screened from the database and another 8 articles were excluded due to bias. Twelve studies were read in extenso and were included in the qualitative analysis. The summary of the studies that met the inclusion criteria is shown in Table 1. The PRISMA flow chart reports the search methodology (Figure 1).

Five studies assessed the benefits of teleassistance in orthodontics for the management of patients at a distance. They all stated that teleorthodontics has the potential to provide significant and determinant help even if further investigation is deemed necessary [19,20,21,22,23].

Five papers evaluated the efficacy and reliability of orthodontic teleassistance in the diagnosis treatment and follow-up of patients [24,25,26,27,28]. 

One study endorsed the use of teleorthodontics for remote patient management [29]. 

One study evaluated which of the available IT technologies would allegedly be used in the near future for remote patient management [30]. All the included studies agreed on the advantages of introducing teleorthodontics into clinical practice. Taking into consideration the 11 included studies, one was not analyzed by MMAT, as this system is not suitable for non-empirical studies such as reviews and theoretical papers [20].

A total of 11 studies were analyzed by MMAT, nine of them were quantitative, two qualitative, and no studies used mixed methods. All the papers that were included were rated equal to or above 50% (average score 79.5%) according to the Mixed Method Assessment Tool and were therefore included. Table 1 presents a detailed summary of each of the 11 studies included.

### 3.1. Available Technologies 

Currently, available technologies that can be used in teleorthodontics are: high-speed Internet connection, digital videos and photographs, smartphones, and websites. A review by Costa et al. [30]. emphasized that peer-to-peer communication services (MSN, Skype, etc.) can be helpful in patient management but that they are not sufficiently reliable by themselves, since they are products of big companies, they may be subjected to unpredictable changes. The authors of the aforementioned review thus recommend using websites instead, as they are easier to use and require no installation. In order to minimize problems involving safety, the same authors recommend using anti-virus and/or firewalls and adopt only sites with valid digital certification and end-to-end data encryption [31]. WhatsApp messenger seems to be the most widely used communication tool according to available literature [29]. Maintaining periodic virtual contacts, while it is impossible to do otherwise, is a valuable tool to build and maintain a positive patient–clinician relationship and a valuable therapeutic allegiance [29].

Digital technology in imaging and impression taking, that is now commonplace in most dental practices, is a powerful tool for the orthodontist to access, analyze and, if need be, communicate with patients, colleagues, and/or dental technicians. The widespread diffusion of smart phones among doctors and patients led to the development of a new option. [32,33] Indeed, an application for smartphones that allows remote monitoring of orthodontic patients using an algorithm of artificial intelligence, has recently been developed. This application is called Dental Monitoring^TM^ (DM) [34]. Its purpose is to provide a precise record of the patient’s occlusion with the integrated phone camera. DM was designed to carry out orthodontic follow-up at a distance. It tracks tooth movement through a 3D reconstruction of an intraoral movie taken with the smartphone camera and specific cheek retractors. The patients themselves make a video that is processed into a scan by DM^TM^. Therefore, orthodontists can perform real-time monitoring of treatment outcomes anywhere and anytime.

This smartphone application (Android, iOS) was originally designed to provide access to orthodontic treatment for people living in places with limited access, to improve comfort and fruibility of the service for people who have busy schedules or travel frequently for work. Similarly, patients who are on orthodontic treatment during the COVID 19 pandemic period can benefit tremendously from remote monitoring, avoiding unnecessary follow-up appointments. Patient monitoring through this simple software may also improve treatment efficacy by avoiding late detections of problems such as debonded brackets, broken ligatures, non-tracking aligners, and are therefore able to solve the problem in the early stage [22]. 

### 3.2. Benefits of Teleorthodontics 

A review on the benefits of teledentistry published in 2018, which considered only papers with high quality assessment scores, stated that not only is teledentistry potentially an effective tool for patient management, but it also has a positive economic impact on the dental profession. This review also pointed out that there is a rapid increase in the number of publications as to the efficacy of teledentistry, especially in oral medicine, periodontics, pediatric dentistry, and orthodontics [20].

However, due to the lack of conclusive evidence and the different methods (outcomes, assessment methods, main goal, etc.) they adopted, the findings cannot be generalized.

Other papers evaluate teleorthodontics as a means of performing initial examinations and report that there was no disagreement between in-office assessment and remote assessment through clinical photographs as to diagnosis and treatment planning [11,21]. They demonstrated that teleorthodontics reduced costs and provided treatment access to a wider range of persons able to benefit from specialist treatment at a distance, without compromising the quality of care [11,35,36].

A study by Favero et al. reported how new technologies applied to orthodontics allowed for remote management of several common orthodontic questions that would have otherwise necessitated in-office treatment: e.g., ligature displacement, discomfort from the appliance, cheek irritation [21]. 

A preliminary study by Hansa et al. [22]. evaluated whether the use of remote monitoring, carried out with DM^TM^ software, is able to reduce the number of in-office visits compared to the traditional appointment management. The same study used a questionnaire to assessed patients’ attitude towards the use of a remote monitoring software during treatment the patients who had remote monitoring had fewer in-office appointments: 1.68 in average during the 7-month follow-up taken into consideration. This means that over a 2-year treatment period, an average of 5.8 in-office appointments could be avoided by the use of DM software. Most patients classified the application as user-friendly (easy or very easy) (86%) and (84%) thought it was useful for their treatment. The questionnaire revealed that most patients using DM had the sensation of enhanced communication with their dentist and better convenience. However, this study [22] provides only preliminary results and, as do other studies, suggests that if the whole treatment period were to be considered instead of just 7 months, more precise information on the effects of teleorthodontics could be obtained. 

Some studies analyzed the benefits of teleassistance in orthodontics and reported their utility in periodic check-ups for those in retention to make an early identification of problems and immediately book in-office appointments, thus maintaining a good doctor–patient relationship and a good level of surveillance over finished cases, without taking up the dentists’ and patients’ time unnecessarily [26].

### 3.3. Effectiveness and Reliability of Proposed Methods

Several studies have described teleorthodontics as an effective tool that allows the orthodontist to maintain treatment control in situations where the patient cannot go to the clinic [37]. These results are in agreement with data reported by Berndt et al. [19]. The authors provided evidence of the viability of teleorthodontics during interceptive treatment. Other studies have shown that the use of new patient monitoring technologies has enabled dental professionals to enhance the quality of treatment provided to their patients, as reported by Mandall and Stephens [23,38]. These authors stated that teledentistry is an effective way to identify appropriate referrals and that teledentistry may well increase treatment efficacy [20].

Dunbar et al. compared the reproducibility of treatment planning performed on digital records, clinical examinations, and standard records. The paper also considered patients’ opinion of in-office visits and teleassistance [25]. It showed that that 50% of the observers were influenced by the type of records used to decide which treatment was more appropriate. The agreement between doctors was higher on standard records than on digital ones. The authors of this study concluded that it is possible to save money, time, and avoid the need to go to the dental office for a consultation [25]. Bradley et al. also made favorable comments about this system [28]. The attitudes toward teleassistance in orthodontics, and in general, dentistry by respective dental care professionals, was investigated in several studies which confirmed it was as an effective alternative to in-office visits for several routine procedures and to make consultations more accessible to dentists and patients [23,38]. Mandall concluded that teleassistance in dentistry is a reliable tool, enabling the screening of new patients and therefore, that it was of substantial help in lowering incorrect referral rates and reduced the waiting list for fist consultations [23].

Morris et al. [24] stated that three dimensional impressions taken with dental monitoring software do not differ greatly to those taken with an intraoral scanner (iTeroElement, Align Technology, Santa Clara, CA, USA). There was a clinically insignificant mean difference of 0.02 mm between the digital models generated with dental monitoring and intraoral scans, suggesting and therefore judging it clinically insignificant [24]. Heather et al. [26] evaluated the reliability and accuracy of DM. They assessed intercanine and intermolar distances during rapid maxillary expansion (RME) on DM scans and on digital models taken at in-office follow-up appointments. The paper reported a slightly higher margin of error for DM scans compared to digital model at the molar level. However, in-office and DM measurements differed by less than 0.5 mm. Therefore, the author of this study concluded that, as long as DM scans are of acceptable quality, they can be reliable in the formulation of clinical decisions. The reliability of DM in the evaluation of rapid palatal expansion treatment, compliance and satisfaction were also studied by Kuriakose et al. [27], who was in agreement with the aforementioned claims. That is, DM was able to make a remote assessment of the condition of posterior crossbite. No significant difference was noted in intermolar width between DM, digital model, or intraoral examination. On the basis of these data, it seems that in-office control of maxillary expansion can be substituted by teleassistance with DM software [27].

Bernd et al. [19] stated that facial orthopedic treatments can be delivered by sufficiently trained general dentists through remote supervision of an orthodontist using teleassistance technology. This may well make a significant improvement in conditions of malocclusion in children, who for various reasons, cannot be treated in-office by an orthodontist. Even if most patients treated with phase I orthodontics usually require a phase II treatment cycle, malocclusion is far less complex and is, therefore, easily managed.

## 4. Discussion 

The ever more powerful capacity of modern computers has led to a continuous development of innovative technologies. Indeed, currently, various branches of medicine and dentistry are benefiting from the advances provided by new technologies for the diagnosis and treatment of several pathologies. Teledentistry is the part of telemedicine that deals with the application of ICT to dental care. Moreover, teledentistry and dental video phoning allow colleagues to readily exchange information. It can also become a cutting-edge screening system able to reduce patients’ waiting time for specialist advice. As long as it is correctly set up, it is capable of improving service and working conditions and may even reduce costs [21]. Teleorthodontics generally refers to any orthodontic care delivered through information technology. A common and relevant example could be that of colleagues being able to discuss the digital records of clinical cases over the Internet and to exchange advice and share experience.

The first studies on teleorthodontics date back to the early 2000s [23,38]. A remarkable example that yielded promising results was a paper investigating the possibility to deliver orthodontic treatments through the remote real-time supervision of an orthodontic specialist for general dentists so as to reach patients with limited access to orthodontic care [19,38]. Another useful application is remote retention check-up by sending images rather than physically going to the dentist [22].

However, most likely, we shall have to wait for yet another decade before teleorthodontics becomes a viable option as technological and cultural obstacles still have to be overcome. 

In recent years, the number of patients who wish to undergo orthodontic treatment requiring fewer in-office visits, while at the same time allowing the specialist to maintain control over the progress of their treatment, has grown. Teleorthodontics as a mean to further reduce unnecessary journeys to the orthodontic practice while maintaining control over treatment is allegedly one of the main reasons teleorthodontics has gained ground over the past few years. The development of clear aligners and lingual custom prescription brackets with robotic multi-wires has significantly reduced chair-side time and in-office visits [39,40]. As a rule, aligners or wires are changed during in-office visits at pre-established appointments that have been made on the basis of personal experience and common knowledge of an approximated time span for the wire to have exhausted its biological efficacy. However, a one size fits all approach is not always ideal, as average values do not take into account a patient’s individual biological response. Teleorthodontics allows for tailor-made scheduled in-office visits though remote monitoring, promoting a more productive workflow. 

These procedures are capable of reducing chair time and improving patient convenience.

As reported in the results of our review, it appears that the most promising technology for teleassistance in orthodontics is Dental Monitoring^TM^ (DM). This is based on three integrated platforms: a smartphone application that takes the patient through correct record taking; a software that adopts an algorithm that quantifies individual tooth movements (less than 0.5° for mesiodistal angulation and faciolingual inclination, and rotation) and an Internet-based interface where the dentist can check patients’ updates as soon as they are uploaded and interact with the patient. Alerts can be set at certain thresholds to receive warnings should an emergency condition arise, e.g., debonded brackets, gingival issues depending on poor hygiene, non-tracking aligners, and so forth and/or for specific treatment objectives. All images recorded by the software are available on the clinician’s platform. Physicians can take advantage of in-office photos (baseline and interim photos) as reference to better understand changes. The software allows four possible monitoring levels, i.e., the number of photos per period of time. Routine pre-treatment monitoring requires one picture every couple of months. The monitoring of active treatment requires one or two photos per week (for aligners and the other therapies respectively). The monitoring of the retaining phase has a more complex picture timing scheme: once weekly for a month, then once monthly for six months, followed by once every couple of months. The last possibility is known as DM Go Live, which is for use in aligner therapy, where pictures are taken once weekly and the patient is informed whether to keep the same aligner or to proceed to the next one. Although expectations are promising, teleassistance in orthodontics could have some limitations. Forwarding scans each 7 days may become a nuisance and frustrating for the patient as they may sometimes need to be taken again. 

Moreover, the reduction in the time spent visiting the patient in person may deteriorate the patient–doctor relationship. Therefore, consent and education are needed for the patient to begin a similar path in order to build a positive and strong therapeutic allegiance [22]. During the pandemic outbreak, orthodontists had to significantly reduce, and in certain cases, suspend follow-up visits of patients currently under active treatments. We can therefore say that the use of applications for monitoring orthodontic therapy could be an effective solution to continue to keep deferrable orthodontic patients under control during the closure of dental practice due to COVID 19 and to reduce unnecessary in-office appointments.

The Italian Society of Orthodontics (SIDO) has recently published the recommended guidelines on the management of orthodontic patients during the COVID-19 outbreak. Orthodontic emergencies are unpredictable issues caused by orthodontic appliances that provoke pain or discomfort, thus requiring urgent dental care [29]. Orthodontic emergencies should be faced using a stepwise approach. The first recommended approach should be virtual assistance through photographic documentation or a video call. It is important to perform a preliminary triage to distinguish situations that require in-office treatment rather than those that are remotely manageable. Unlike other dental questions, orthodontic problems like traumatic injuries of teeth and periodontal structures, abscesses, etc., have a lower degree of severity and often do not necessitate in-office care to be solved. The most common orthodontic emergencies are related to the detachment of one or a few brackets and acute stinging of the lips and the oral mucosa caused by orthodontic wire or scraping brackets. Many of these problems can be readily solved at home with less stress for patients’ families, saving time for both patients and dentists alike. Since they are not true emergencies, they can, more often than not, be easily resolved by providing the patient with simple instructions during a video call or with typed messages after photographic documentation of the problem, describing the intraoral condition. It has been suggested that dental caregivers become familiar with the potential social networks and modern web-based communication platforms have, thanks to the possibility of making a precise evaluation of indications and contraindications [41,42]. Patients should continue ongoing therapies, but should also be video checked periodically. Dental professionals and their team must select the number of eligible patients and organize this procedure [29]. In all other cases, it is advisable to contact each individual patient in therapy actively in order to give specific indications and it is recommended to make telephone appointments with patients 4–6 weeks apart to carry out a further check-up or fix an appointment in the studio, if strictly necessary [29]. Patients must be reassured and periodically checked, in particular if they have discomfort or problems related to their orthodontic appliance.

It is important to emphasize that not only must emergencies be managed by the orthodontist, but also all other patients with both mobile and fixed appliances. Teleorthodontics relies on information technology and telecommunications and allows for various types of orthodontic follow-up visits at a distance. Therefore, it is of fundamental importance to be able to avail oneself of teleorthodontics for the constant monitoring of all patients should the orthodontic practice be shut-down and/or visits significantly limited.

The professional, thanks to special devices, can check a patient’s real situation remotely and compare it with the digital setups previously made, especially in the case of treatments with lingual orthodontics and aligners [26]. Based on the revised articles, new information technology improved the management of orthodontic patients, and in numerous cases, allowed for their remote management. Teleorthodontics has the potential to improve patient management and reduce treatment costs.

This review does have some limitations. One such limitation is the fact that the studies included had only a fair scoring in MMAT. Moreover, although most papers reported a positive attitude towards teleorthodontics, a publication bias may be present since all papers reported one or more positive outcomes for accuracy and/or efficacy and some papers are technical reports or pilot studies. 

Only a limited number of papers made a controlled comparison of teleassistance in orthodontics with traditional methods. Many included studies focused on the evaluation of efficacy rather than the effectiveness of teleorthodontics. It would be of great importance to evaluate the appropriateness of teleassistance in orthodontics by assessing clinical outcomes and costs/template details the sections that can be used in a manuscript. 

## 5. Conclusions

This review found a growing number of studies sustaining the efficacy of teleassistance in orthodontics. The advent of a large number of technological innovations over the past few years in dental and in orthodontic practices has allowed for substantial improvement. The COVID-19 pandemic will surely have long-term effects on patient management as it seems unlikely there will be any definitive treatment or vaccines available in the near future. This condition will require a different organization of dental appointments for several months to come and remote patient management could be efficiently carried out using instant messaging platforms to deliver healthcare consultancies. We believe that teleassistance in orthodontics should be considered a welcome resource, as it is able to successfully manage many dental emergencies, to reassure and follow patients at a distance without exposing them and/or dental practitioners to unnecessary risks. Moreover, most case issues can be promptly solved without the patients coming to the orthodontist office by communicating with photos and/or videos, saving both the patient’s and clinician’s valuable time.

The aim of teleorthodontics is fulfilled by reducing unnecessary follow-up visits while maintaining regular monitoring, thus not jeopardizing expected results. The potential of teleorthodontics is virtually endless; remote consultations could be carried out across the globe without the obstacles of distances or of scheduling appointments. This kind of approach could be of great help in the management of all dentofacial orthopedic removable appliances and of orthodontic treatments that need little in-office maintenance, such as some clear aligner therapies. 

Even though in-office visits are still required for many dental and orthodontic procedures, teleorthodontics opens up open new horizons in the treatment and follow-up of many patients.

Nevertheless, currently, most studies report only pilot studies and evaluate short-term results of teleassistance in orthodontics. Therefore, there is limited evidence and the study designs differ, as do the interventions and endpoints assessed in the papers included, meaning that our findings cannot be strictly generalized. However, although we are of the opinion that further studies with higher levels of evidence are needed to objectively evaluate efficacy, cost-effectiveness, and long-term results, we are confident that teleassistance in orthodontics will have a role to play in the future.

## 6. Future Implications 

Technological advances have substantially changed dentistry. In orthodontics, software like Dental Monitoring, which is capable of providing web-based platforms for sharing health data [43] between patients and doctors, may allow orthodontists to closely monitor their patients’ status, reducing in-office visits and delivering more patient-centered treatment. The aim of teleassistance in orthodontics is to reduce unnecessary in-office visits and improving monitoring and early treatment of problems that may jeopardize the desired final outcome. Teleassistance in orthodontics may allow for remote consultations that can be carried out wherever without the need for the patient to be anywhere near the office. The downside of teleorthodontics is the reduced time to develop and maintain a positive relationship between doctor and patient [44,45].

Dunbar [25] reported in a feasibility study that 70% of patients thought that in-office consultation was extremely important, and most patients preferred it to teleorthodontics assistance. 

Another important consideration are the points of law regarding patient confidentiality that may be in danger because of digital communication of sensible data over the Internet [46]. The quality of the doctor–patient relationship is particularly important should the treatment have complications or if outcomes are deemed unacceptable. Malpractice lawsuits may increase if patients feel they are not receiving treatment of a satisfying quality. Moreover, teleassistance in orthodontics and patients’ confidentiality issues are more complicated in orthodontics because patients are often minors. 

Therefore, the teleorthodontics we are implementing during the COVID 19 pandemic should not be seen in the future as a new treatment option for patients looking for cheaper and aesthetic alternatives to traditional orthodontics, as dental and orthodontic care should not be reduced to a simple question of “commodity” [47]. As already reported by Hansa et al. [22], orthodontists are still a little doubtful about treatment at a distance because of the possibility of limiting their patient base and due to the risk patients may face. It is the authors’ opinion that, in the future, teleassistance in orthodontics will be helpful to maintain high standards of care while reducing unnecessary in-office visits in order to improve rather than reduce the quality of the service already provided by conventional orthodontics.

## Figures and Tables

**Table 1:** Description of included studies and Mixed Methods Appraisal Tool (MMAT) score.

Author	Year	Study Design	Journal	Primary End Point	MMAT Score and Study Limitations
Morris R.S et al.	2019	Cross-sectional study	Am J Orthod Dentofacial Orthop	Digital models taken with Dental Monitoring software (DM) are precise enough for clinical application.	MMAT score 75%. The sample is too small to extend to the entire population.
Costa A.L.P. et al.	2011	Qualitative	Dental Press J. Orthod	Remote management of clinical cases was successfully carried out. Therefore, teleorthodontics is deemed a viable option. Available technologies, that are accessible and reduced in cost, suggest a quick development of teleassistance in orthodontics in the near future.	MMAT score 100%.
Bradley S.M et al.	2007	Survey	Primary dental care	Almost 50% of the responding dentists working in primary care in this area had a positive attitude towards a teledentistry-based referral scheme to the orthodontic consultants. These practitioners were more likely to be familiar with the use of digital camera and using removable appliances.	MMAT score 100%.
Estai M et al.	2018	Systematic review	J Telemed Telecare	There is emerging evidence as to the efficacy of the use of teledentistry technologies. However, there is some doubt about the price and the long-term efficacy. As the current evidence is inconclusive, further research is needed.	Not applicable
Favero L. et al.	2009	Survey	European Journal of Paediatric Dentistry	Teleassistance in dentistry is a new and powerful tool that makes for effective communication between the care provider and patient and between the providers themselves. The study stated that these technologies can be of significant help in treating orthodontic emergencies.	MMAT score 75%. The sample is not representative of the target population.
Dunbar A.C. et al.	2014	Prospective observational cross- sectional study	Health Eng.	Observations taken from this pilot study through the assessment of treatment planning and comparison between patients’ opinion about traditional dental examinations (face to face) and teleorthodontics showed that the treatment planning was influenced by the diagnosis of the observer. However, the consultation system satisfied both the clinician and the patient.	MMAT score 50%. A bigger sample of patients should have been analyzed to evaluate the significance of differences in treatment planning because of the three diagnostic means. Most patients came from urban areas and only one center was considered. Concordance between operators in treatment planning was not possible to be assessed completely because not all the observers examined every subject clinically.
Hansa I. et al.	2018	Survey	Seminars in Orthodontics	This preliminary study assessed the possibility to reduce the number of in-office visits using teleorthodontics software. The patients’ feedback on the use of the aforementioned software was positive.	MMAT score 100%.
Moylan H.B. et al.	2019	Cross-sectional study and survey	Angle Orthodontics	A comparison between measurements performed on models taken in-office or with Dental Monitoring software. Intercanine and intermolar distances showed little difference (below 0.5 mm). Therefore, this orthodontic teleassistence system is reliable to make clinical decisions.	MMAT score 100%.
Berndt J. et al.	2008	Case-Control Study	Am J Orthod Dentofacial Orthop	Children presenting malocclusions in remote areas with difficulties in accessing orthodontic care could get interceptive orthodontic treatment through cooperation between general dentists and orthodontists using teledentistry.	MMAT score 75%. It is unclear whether all the outcome data have been provided.
Mandall N.A. et al.	2005	Randomized Controlled Trial	Br Dent J	The authors deemed that teledentistry is an effective method to identify appropriate referrals. Moreover, they stated that teledentistry could increase treatment effectiveness.	MMAT score 100%. No limitations.
Kuriakose P. et al.	2019	Cross-sectional study and survey	Journal of the World Federation of Orthodontists	Dental Monitoring is a reliable tool to monitor rapid palatal expansion in the correction of the crossbite. Dental Monitoring, digital impression, and in-office examination showed no significant difference in assessing intermolar distances. The study concluded that in-office assessment of a rapid maxillary expander can be successfully substituted with teleorthodontics (DM software).	MMAT score 50%. This paper presents a small sample size due to several dropouts. The sample was selected from a pool of patients attending a single hospital and, therefore, the conclusions cannot be extended to a private setting.
Caprioglio A. et al.	2020	Qualitative	Progress in Orthodontics	Teleorthodontics is a useful tool to manage emergencies, and monitor patients at a distance using WhatsApp.	MMAT score 50%. The study is based on personal experience. No patients were analyzed, no results were evidenced
